# Identification and Classification of Fungal GPCR Gene Families

**DOI:** 10.3390/jof12010030

**Published:** 2025-12-30

**Authors:** Zhiyin Liu, Asaf Salamov, Igor V. Grigoriev

**Affiliations:** 1 U.S. Department of Energy Joint Genome Institute, Lawrence Berkeley National Laboratory, Berkeley, CA 94720, USA; lisaliu@lbl.gov (Z.L.); aasalamov@lbl.gov (A.S.); 2 Department of Plant and Microbial Biology, University of California Berkeley, Berkeley, CA 94720, USA

**Keywords:** fungal genomics, G protein-coupled receptors, signaling, protein structure

## Abstract

G protein-coupled receptors (GPCRs) are transmembrane proteins crucial for signal transduction in eukaryotes, responding to diverse extracellular signals. Researchers have found and systematically summarized 14 distinct types of GPCRs in fungi but their distribution among numerous fungal species remained largely unexamined. Additionally, three families of mammalian homologs (Rhodopsin, Glutamate, and Frizzled) have been found in previous studies, but they are not included in the systematic classification of fungal GPCRs. Our study establishes a unified classification of 17 GPCR classes in fungi, combining 14 fungal and 3 mammalian previously recognized groups, and classifies 28,294 GPCRs across 1357 fungal species, significantly expanding the scale of GPCRs in fungi and demonstrating their broader distribution. We found that mammalian homologs are notably more prevalent in Early Diverging Fungi (EDF), whereas the previous 14 classes are predominantly found in Ascomycota and Basidiomycota. The most abundant class detected in fungi was Pth11-like GPCRs, exclusively found in Pezizomycotina and involved in fungal pathogenicity. Our analysis suggested that Pezizomycotina ancestor possessed an extensive array of Pth11-like GPCRs, but over time, some species underwent considerable reductions in these GPCRs in conjunction with genome contractions. Utilizing a custom-built convolutional neural network (CNN) for the identification of fungal GPCRs, we identified several putative novel fungal GPCRs. Predicted interactions between these prospective new GPCRs and G-alpha proteins, as simulated by AlphaFold Multimer, provided additional support for their functional relevance. In conclusion, our work defines the first large-scale, unified classification of fungal GPCRs, reveals lineage-specific expansions and contractions, and uncovers previously unrecognized GPCR candidates with potential functional roles in fungal signaling.

## 1. Introduction

G protein-coupled receptors (GPCRs) are a large and diverse family of membrane-bound signaling proteins with seven transmembrane helices (TMHs). In mammals, these receptors mediate a plethora of extracellular inputs into the cell and regulate several biological processes including vision, taste, neuroendocrine functions, and immunity [1,2]. In fungi, GPCRs are largely utilized in sensing the environment, sexual development, pathogenesis, and other critical functions [3,4,5,6]. Their ability to detect external stimuli is particularly important for fungal survival and adaptation to habitats that are often characterized by nutrient limitation and environmental stress [7,8]. Despite the extensive characterization of GPCRs in mammals, their counterparts in fungi remain comparatively underexplored and only a fraction of mammal diversity has been mirrored in fungal species [3].

To date, fungal GPCRs have been systematically classified into 14 distinct classes [9]. The first five classes are Ste2 pheromone receptors [10], Ste3 pheromone receptors [10], glucose-sensing Gpr1 homologs [11], nutrient sensors analogous to Stm1 in *Schizosaccharomyces pombe* [12], and cAMP receptors homologous to those in *Dictyostelium discoideum* [13]. These receptors activate two key signaling pathways, the mitogen-activated protein kinase (MAPK) cascades and the cAMP-dependent protein kinase A (PKA) pathway, which are essential for fungal physiology [14]. Several of these GPCR classes have well-characterized and conserved ligands: Class 1 receptors detect the α-factor peptide pheromone [15], Class 2 receptors recognize the a-factor peptide pheromone in *Saccharomyces cerevisiae* [16], and Class 3 receptors function as conserved glucose sensors across yeast species [11]. GPCR Class 9 is microbial opsins [17]. In addition, eight fungal GPCR classes discovered later include GprK-like receptors with RGS domains [18], receptors similar to the rat growth hormone-releasing factor receptor [19], mPR-like/PAQR receptors [20], the Lung 7TM superfamily [21], GPCR89/ABA receptors [22], Family C-like receptors [23], the DUF300 superfamily/PsGPR11 [24], and Pth11-like receptors [19]. Notably, GPCR89/ABA receptors (Class 11 GPCRs) were found to possess 9 TMHs instead of 7 [23].

Despite these advances, our current knowledge of fungal GPCRs remains restricted to a limited number of species, and their relationship to mammalian GPCR families is still poorly resolved. Mammalian GPCRs are grouped into five major families: Glutamate, Rhodopsin, Adhesion, Frizzled/Taste2, and Secretin, collectively known as the GRAFS classification system [25]. Of these, only Class 7 includes the Pfam domain 7tm_2 homologous to the mammalian Secretin GPCR family. However, three classes of mammalian homologs (Rhodopsin, Glutamate, and Frizzled) detected in previous studies [26], were not included into the existing 14-class fungal framework. This presents an opportunity to expand the fungal classification by formally incorporating these three mammalian homologous classes, increasing the total to 17 classes, and therefore to provide a more comprehensive classification of fungal GPCRs.

Among the several types of GPCRs, Pth11-like GPCR has been identified as key regulators of late endosomal homotypic fusion and protein sorting in *Magnaporthe oryzae*, influencing appressorium growth, cAMP generation, and ultimately the pathogen’s virulence [27]. Notably, Pth11-related GPCRs were only found in Pezizomycotina [19,27,28]. Pth11 contains an amino-terminal extracellular cysteine-rich CFEM domain (pfam05730), which is involved in diverse fungal processes, including pathogenesis in *Magnaporthe grisea* and *Candida albicans*, as well as cell wall biogenesis and stability in non-pathogenic fungi such as *S. cerevisiae*. However, the CFEM domain was found only in a small number of Pth11-like proteins from *M. grisea* and *Neurospora crassa* [29]. The abundance of Pth11-like GPCRs across species remains unknown, despite their extensive distribution in Pezizomycotina.

Due to the diverse functions and evolutionary divergence of GPCRs, predicting their presence and roles in different fungal species is challenging. Recent improvements in deep learning, particularly in protein function prediction, present new opportunities. Recent advancements in natural language processing and sequential data preprocessing have enabled the application of deep neural networks (DNN), convolutional neural networks (CNN), recurrent neural networks, long short-term memory networks, and transformer-based models [30]. Furthermore, features derived from amino acid sequences and three-dimensional (3D) protein structures can be incorporated into multi-modal deep learning techniques. Approximately half of protein function prediction tools employ 1D CNNs combined with DNNs, effective for sequence classification [31], for example, ProteInfer [32] and DeepFRI [33]. ProteInfer utilizes deep CNN to predict Enzyme Commission numbers and Gene Ontology terms from unaligned amino acid sequences. Conversely, DeepFRI employs a graph convolutional network that integrates characteristics from a protein language model and structural data.

A defining feature of GPCRs is their ability to activate G-proteins, making protein–protein interaction (PPI) prediction critical. Machine learning-based models, such as D-script [34], increasingly complement traditional experimental approaches. Advances in 3D structure prediction, especially via AlphaFold Multimer [35], have improved prediction of interactions within protein complexes.

In this study, we analyzed 1357 fungal species, and provided a comprehensive view of GPCR diversity and distributions among species. Additionally, we examined the distribution of Pth11-like GPCRs in Pezizomycotina and their evolutionary dynamics within the class Sordariomycetes, and potential links to host–pathogen interactions and secondary metabolism. We predicted novel GPCRs featuring seven TMHs using a one-dimensional CNN binary classifier specifically designed for the identification of fungal GPCRs. Supported by DeepFRI and ProteInfer, and followed by modeling interactions between candidate GPCRs and G-alpha proteins using AlphaFold Multimer, we identified high-confidence GPCR–G-alpha combinations for further validation.

## 2. Materials and Methods

### 2.1. Identification of Putative GPCRs Across Fungal Species Using Known Fungal GPCR Classification

In this analysis, 67 GPCR query sequences, spanning all 14 recognized classes, were subjected to BLASTP 2.8.1+ [22] searches against genomes of 1357 species from the MycoCosm database [36]. Searches were constrained to an E-value < 1 × $10^{-5}$ and required hits to cover >75% of both the query and MycoCosm protein entries. The number of TMHs of the candidate GPCRs were verified using TMHMM [37], retaining only sequences with 6–7 TMHs, to account for TMHMM’s limitations in accurately determining helix counts. For Class 9 GPCRs, we maintained the sequences with 7 to 9 TMHs because Class 9 GPCRs were originally found with 9 TMHs [23]. Multiple sequence alignments were produced with MAFFT version 6.717 [38]. Then regions lacking conservation were excised using trimAl [39]. Hidden Markov Models (HMMs) were constructed for 14 GPCR classes using ‘hmmbuild’ from HMMER 3.2 [40], and ‘hmmsearch’ was employed against publicly available proteomes from MycoCosm database to identify potential homologs with an E-value threshold of <1 × $10^{-5}$. Sequences retrieved from ‘hmmsearch’ were re-evaluated using TMHMM, and only those matching the TMH criteria were retained. Sequences whose TMHMM-predicted topology did not exhibit the expected extracellular N-terminus and intracellular C-terminus were further filtered out [41]. Finally, Pfam domain identification isolated sequences containing fungal GPCR-functional domains (Table 1), thereby delineating a subset of putative GPCRs within a diverse array of fungal species.

### 2.2. Identification of Fungal Homologs of Mammalian GPCRs

Functional Pfams from each mammalian GPCR family include 7tm_1 (Rhodopsin), 7tm_3 (Glutamate), Frizzled/Fz (Frizzled), TAS2R (Taste2), and GAIN/GPS/hEGF/EGF_CA/ Calx-beta/Cadher (Adhesion). These were identified in 1357 fungal species, and hits with a *p*-value < 1 × 10^−5^ containing these Pfam domains were verified using TMHMM [37], retaining only sequences with 7 TMHs and expected topology (extracellular N-terminus, intracellular C-terminus) [41].

### 2.3. Comparative Phylogenetic Analysis of Pth11-like GPCRs

Phylogenetic trees have been constructed for a specific clade of species within Pezizomycotina (Supplementary File S1 Table S6) that are closely related and exhibit substantial variations in the abundance of Class 14 GPCRs. Initially, we conducted a search for species of interest within the Pezizomycotina species tree available on the JGI genome portal [36]. Ultimately, we identified 12 species (Table 2) that share close evolutionary relationships. These species also had more than 10 times the abundance of Pth11-like GPCRs compared to other species. The species tree for the 12 genomes and the gene tree for Pth11-like GPCRs were built using OrthoFinder version 2.5.4 [42]. Both trees were visualized and compared using iTOL version 6.9.1 [43].

### 2.4. Comparative Gene Families Enrichment Analysis

OrthoFinder was employed to identify orthogroups in species exhibiting substantial differences in Pth11-like GPCR abundance. Notung version 2.9.1 [44] was used to infer duplication and loss events for Pth11-like GPCRs. To detect expanded and contracted gene families, we applied CAFE5 [45], filtering significantly expanded or contracted families using a threshold of p<0.05. Gene Ontology (GO) enrichment analysis was executed using TBtools-II [46] and Pfam domain enrichment analysis were performed on these gene families, and statistical significance was assessed using a two-sided test with *p*-values adjusted via the Benjamini–Hochberg method [47].

### 2.5. Potential Novel GPCR Prediction

#### 2.5.1. 1D-CNN Architecture

We implemented a 1D Convolutional Neural Network (1D-CNN) to classify protein sequences as either GPCR or non-GPCR. Positive data consists of all sequences from all 14 classes of GPCRs that we identified. For the negative dataset, we collected 18,221 sequences containing 7 TMHs, with Pfam domains associated with protein functions unrelated to GPCR activity (Table 1). The sequences were one-hot encoded, converting each amino acid into a binary vector, and padding was applied to manage variable sequence lengths. Then the data was split into 80% for training and 20% for testing. The model architecture consists of a 1D convolutional layer with 32 filters, a kernel size of 3, and ReLU activation. This is followed by a max-pooling layer with a pool size of 2, which reduces the dimensionality of the feature maps. The flattened output is passed through a fully connected layer with 64 neurons, also with ReLU activation [48], and a sigmoid-activated output layer for binary classification. The model was compiled using the Adam optimizer [49] and binary cross-entropy loss, and its performance was evaluated based on accuracy. After training for 10 epochs with a batch size of 32, the model achieved a test accuracy of 0.97.

#### 2.5.2. Preparation of Query Sequences

First, we searched all 1357 fungal genomes in the database for sequences with 7 TMHs using TMHMM [37]. The 26,205 newly identified GPCRs, along with 5718 sequences containing 7 TMHs but invalid topologies, were filtered out. Next, MCL clustering [50] was performed. For the largest cluster, multiple sequence alignment was conducted using MAFFT [38] to assess sequence similarity. Sequences from the largest subcluster containing the DUF6534 domain were analyzed to determine whether they represent a novel GPCR.

### 2.6. GPCR—G-Protein Interaction Prediction

#### 2.6.1. GPCR—G-Alpha Protein Interaction Prediction

Well-known GPCR and G-alpha pairs, along with non-interacting pairs, were modeled using Alphafold Multimer version 2.2 [35]. Potential novel GPCRs from 155 species were then paired with G-alpha proteins from the same species and processed through AlphaFold Multimer. The pairs with the highest confidence scores in each species were selected, and the corresponding .pdb and .pkl files were collected for pDockQ2 score calculation [51].

AlphaFold Multimer has been utilized to validate protein complexes, whereas methods such as pDockQ2 [51] evaluate interface quality through predicted aligned error (PAE) and predicted Local Distance Difference Test (pLDDT) [52] metrics in the absence of native structures.

#### 2.6.2. GPCR—G Protein Heterotrimer Interaction Prediction

A total of 411 possible pairings of GPCR and G protein heterotrimers across 10 species with the highest confidence scores from the GPCR–G-alpha protein combinations were modeled using AlphaFold Multimer version 2.2. For each modeled structure, we computed pTM, ipTM, pLDDT, and pDockQ2 scores to assess structural quality and interaction confidence.

## 3. Results

### 3.1. Distribution of Known Fungal GPCR Classes in 1357 Fungal Species

**Figure 1:**
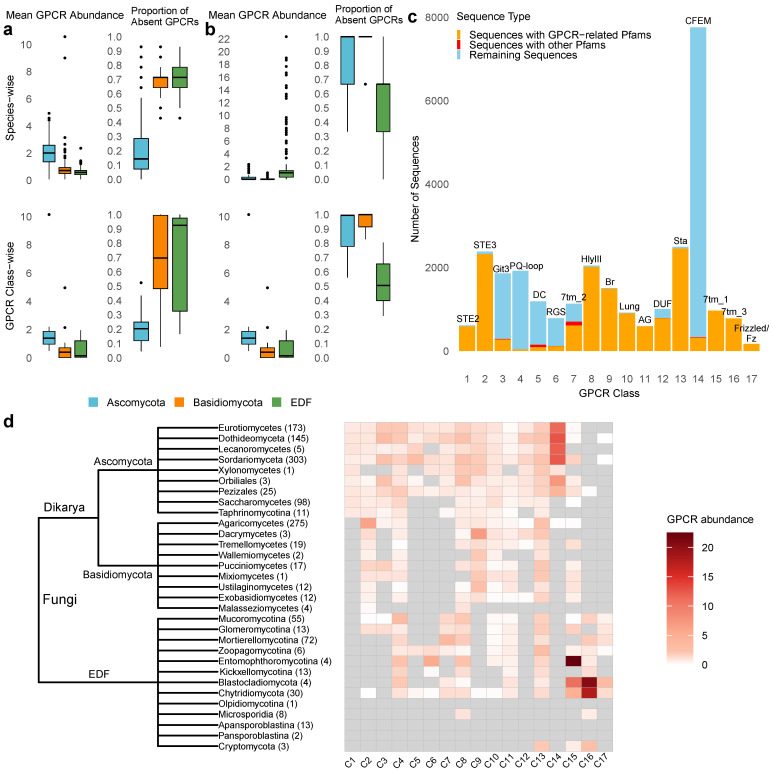
GPCR distribution among 1357 fungal species. (**a**) Box plots showing the mean GPCR abundance and proportion of absent GPCRs across fungi and 14 known fungal GPCR classes. (**b**) Box plots demonstrating the mean GPCR abundance and proportion of absent GPCRs across fungi and 3 mammalian GPCR classes. The settings of the figure are the same as in (**a**). (**c**) Pfam domain distribution across all the 17 GPCR classes. The x-axis represents each GPCR class, while the y-axis shows the number of sequences in each class. Bars are color-coded: the orange section indicates sequences containing the specified GPCR-related Pfam domain (displayed above each bar), the red section represents sequences with other non-GPCR Pfam domains, and the blue section represents sequences without Pfam domains. DC in Class 5 refers to Dicty_CAR, Br in Class 9 refers to Bac_rhodopsin, Lung in Class 10 refers to Lung_7-TM_R, AG in Class 11 refers to ABA_GPCR, DUF in Class 12 refers to DUF3112, and Sta in Class 13 refers to Solute_trans_a. (**d**) Distribution of GPCRs across 31 fungal clades within three groups. The tree illustrates the taxonomic cladogram of fungal classes. The adjacent heatmap displays the average GPCR abundance for species in each fungal class. Numbers following the fungal class names at the tree nodes indicate the number of species within each taxonomic group.

Using 67 literature-derived GPCRs from 14 known fungal GPCR classes (Supplementary File S1 Table S2) queried against 1357 fungal genomes from the MycoCosm database, we found 26,205 potential GPCR sequences across three fungal groups: Ascomycota, Basidiomycota, and the Early Diverging Fungi (EDF) (Supplementary File S2). Significantly, no GPCRs from 14 known fungal classes were detected in 30 species (Supplementary File S1 Table S3). Ascomycota demonstrated the greatest average abundance and diversity among species and GPCR classes, accounting for 79.1% of predicted fungal GPCRs. Conversely, species within Basidiomycota and EDF exhibited many absent or reduced GPCRs at both the species and GPCR class levels in comparison to Ascomycota (Figure 1a).

Further classification of the fungal species into 31 clades showed that species from three groups, Olpidiomycotina, Apansporoblastina, and Pansporoblastina, completely lacked GPCRs across all 14 recognized categories (Figure 1d). Olpidiomycotina is a subphylum comprising a category of obligate parasitic fungi. Apansporoblastina and Pansporoblastina are classified within the phylum Microsporidia, comprising spore-producing unicellular parasites. Furthermore, Class 1 GPCRs, represented by STE2-like fungal pheromone receptors, are exclusively found in Ascomycota, whereas Class 2 GPCRs, which include STE3-like fungal pheromone receptors, are predominantly present in both Ascomycota and Basidiomycota. To be specific, Agaricomycetes in Basidiomycota exhibited the highest abundance of Class 2 GPCRs compared to other GPCR classes and fungal groups. Class 5 (cAMP receptor-like), Class 6 (GprK-like receptors with RGS domains), and Class 7 (rat growth hormone-releasing factor receptor homologs) are exclusively found in Ascomycota and EDF, but are absent in Basidiomycota. Furthermore, Dacrymycetes were characterized by an expansion of Class 9 GPCRs, rhodopsin-like receptors predominant in Ascomycota and Basidiomycota. Pth11-related GPCRs (Class 14), exclusively present in the Pezizomycotina subphylum of Ascomycota, are the most abundant class (29.6%) across all fungi (Figure 1d). This finding expands on previous studies that identified Pth11-related GPCRs as unique to 20 species of Eurotiomycetes and Sordariomycetes in Pezizomycotina [29], to 659 species, now including species from Dothideomycetes, Lecanoromycetes, Xylonomycetes, Orbiliales, and Pezizales.

Pfam domain characterization was used for additional validation of these sequences, where 12,772 out of 26,205 sequences contained specific Pfam domains, with 12,587 (98.6%) of them possessing functional GPCR domains that were consistent with their associated GPCR class (Table 1). While classes 4 and 6 showed the smallest number of sequences with their respective domains, PQ-loop and RGS, Class 8 GPCRs displayed the highest number of sequences including GPCR-related Pfam domain (HlyIII). Furthermore, although Class 14 GPCRs were the most plentiful among fungal species, only 4.1% of these sequences included the CFEM domain, which is linked to fungal pathogenicity in Pth11 GPCRs (Figure 1c). This suggests that the CFEM domain is not inherently essential for the functionality of Pth11-like GPCRs in fungi.

### 3.2. Identification of Mammalian GPCR Homologs in Fungal Species

To identify mammalian GPCR homologs in fungi, we searched for functional Pfam domains corresponding to five human GPCR classes (Rhodopsin, Glutamate, Frizzled, Taste2, and Adhesion) across 1357 fungal species. After filtering for sequences containing seven TMHs, we identified 2089 mammalian GPCR homologs in 594 fungal species, distributed across three mammalian GPCR families: Rhodopsin, Glutamate, and Frizzled, which we suggest to add to fungal GPCR classification as classes 15, 16, and 17, respectively (Figure 1c,d; Supplementary File S1 Table S3; Supplementary File S2). Interestingly, the Adhesion and Taste2 classes appear to be absent in fungi.

Unlike the 14 fungal GPCR classes, which are predominantly enriched in Ascomycota, mammalian GPCR homologs are primarily found in EDF. EDF species have a higher average number of mammalian homologs per species and display a substantially fewer missing or decreased GPCRs at both the species and GPCR class levels, compared to Ascomycota and Basidiomycota (Figure 1b). The majority of mammalian homologs were found in the Rhodopsin class (Class 15), with 984 sequences (47.1%), followed by 918 sequences (43.9%) in the Glutamate class (Class 16), with the remaining 187 (8.9%) in the Frizzled class (Class 17) (Figure 1d). Nearly no Glutamate and Frizzled homologs were found in Ascomycota and Basidiomycota. Large number of Rhodopsin homologs were found in Entomophthoromycotina, a subphylum containing arthropod pathogens and soil- and litter-borne saprobes. Glutamate homologs are enriched in Blastocladiomycota and Chytridiomycota, phyla of fungi characterized by their production of zoospores with a single posterior flagellum often found in aquatic habitats, acting as parasites of plants. In summary, we identified mammalian GPCR homologs from four distinct classes, including the Secretin class, which shares the 7tm_2 domain with Class 7 in fungal GPCRs. This demonstrates some evolutionary conservation of key GPCR families between mammals and fungi, especially in EDF.

### 3.3. Comparative Analysis of Pth11-like GPCRs

**Figure 2:**
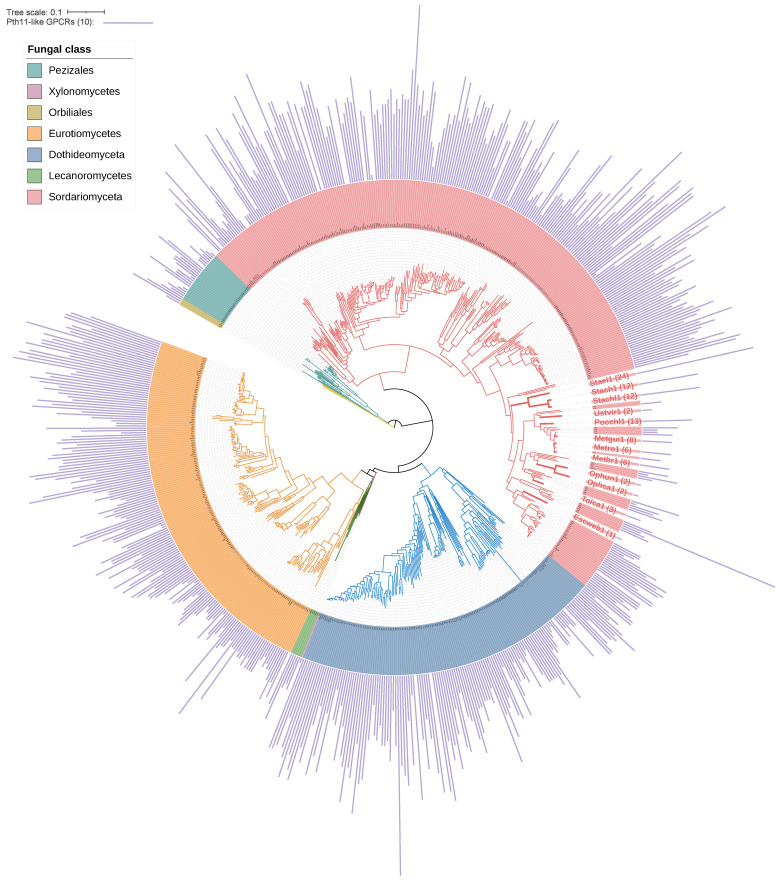
Phylogenetic tree of Pth11-like GPCRs across 640 species from five fungal classes and two orders (Orbiliales and Pezizales) within Pezizomycotina. Species from different classes are color-coded according to the legend. Twelve species from Sordariomyceta are highlighted in bold with enlarged font (Table 2), selected for their phylogenetic proximity and notable variation in Pth11-like GPCR abundance. The number in parentheses indicates the abundance of Pth11-like GPCRs. The purple bar on the outside of the circle represents the abundance of Pth11-like GPCR in each species, varying from 1 to 40 in individual genomes.

The Pezizomycotina subphylum, comprising 640 species with sequenced genomes, exhibits a significant prevalence of Pth11-like GPCRs (Class 14) relative to other GPCR classes, accounting for two-thirds of all fungal GPCRs. Nonetheless, these GPCRs, found in seven fungal groups within Pezizomycotina, display variable abundance (Figure 2), up to tenfold. In order to examine the evolutionary relationships of Pth11-like GPCRs among closely related Pezizomycotina species that exhibit significant variations in receptor abundance, a comparative phylogenetic analysis of 12 selected representative species of Sordariomycetes (Table 2) was conducted.

Initially, we examined the duplication and loss of Pth11-like GPCRs among these 12 species utilizing Notung [44], uncovering 42 duplication and 108 loss events. *Escovopsis weberi*, which is a specialized parasite of the fungal cultivar *Leucoagaricus gongylophorus* [53], demonstrated the highest losses of Pth11-like GPCRs (Table 2). Species with a high abundance of GPCRs typically experienced fewer losses and more duplications. This indicates that the ancestor for these species already had high number of Pth11-like GPCRs. We further analyzed genome expansion and contraction across these species using CAFE [45]. *E. weberi*, *Tolypocladium capitatum*, and *Ustilaginoidea virens* demonstrated a significant number of contracted gene families, accompanied by a comparatively lower number of expanded families. Conversely, species abundant in Pth11-like GPCRs, including *Pochonia chlamydosporia* and *Stachybotrys elegans*, demonstrated a higher number of expanded gene families. This suggests a correlation between the expansion or contraction of Pth11-like GPCRs and the overall dynamics of the genome. The genome size contraction of species with a low abundance of Pth11-like GPCRs may be linked to alterations in their lifestyle.

**Figure 3:**
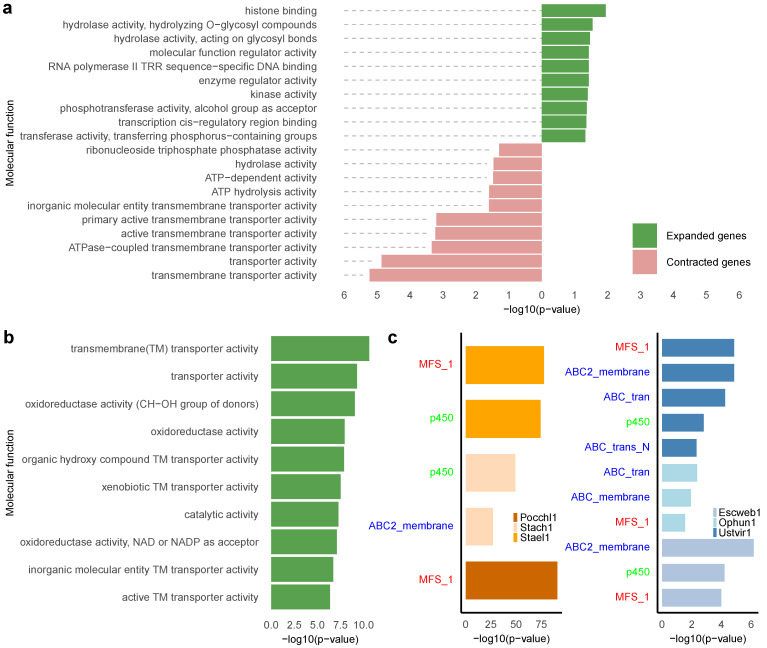
Functional enrichment and Pfam domain analysis across fungal species based on contracted and expanded gene families identified using CAFE. (**a**) Top ten molecular functions for both significantly expanded and contracted genes in the GO enrichment analysis for *Escovopsis weberi*. (**b**) Top ten molecular functions for significantly expanded genes in GO enrichment analysis for *Stachybotrys elegans*. No significantly contracted genes were identified in *Stachybotrys elegans*. (**c**) Pfam domain enrichment analysis: The left panel shows the top significantly expanded Pfam domains in *Pochonia chlamydosporia* (Pocchl1), *Stachybotrys chartarum* (Stach1), and *Stachybotrys elegans* (Stael1). The right panel shows the top significantly contracted Pfam domains in *Escovopsis weberi* (Escweb1), *Ophiocordyceps kimflemingae* (Ophun1), and *Ustilaginoidea virens* (Ustvir1). Common Pfam domains across species are highlighted in red, blue and green. A two-sided *t*-test was performed to identify significant expanded or contracted genes, with *p* < 0.05 considered significant. The Benjamini–Hochberg method was used to adjust *p*-values for multiple testing.

Gene Ontology (GO) analysis was performed on all genes, excluding GPCRs, across the six species that exhibit a Pth11-like GPCR abundance difference of no less than six-fold. In *E. weberi*, significantly expanded genes (p<0.05) were enriched in functions related to gene expression regulation and enzymatic activity modulation (Figure 3a). On the other hand, contracted genes were predominantly linked to transmembrane transport and hydrolase activity. Comparable enrichment patterns were noted in *T. capitatum* and *U. virens*. In contrast, *S. elegans* displayed expansion in genes involved in transmembrane transport and oxidoreductase catalytic activity, with no significant contraction in overall gene sets (Figure 3b). Similar results were noted for *P. chlamydosporia* and *Stachybotrys chartarum*.

Additionally, Pfam domain enrichment analysis indicated that the contracted genes were predominantly enriched in the MFS_1 domain in *E. weberi*, *T. capitatum*, and *U. virens* (Figure 3c). The MFS (major facilitator superfamily) comprises a large group of plasma membrane proteins that function as transmembrane transporters for a wide range of substances, and these transporters are both abundant and diverse in fungi [54]. These three species also exhibited gene contraction in domains related to ABC (ATP-binding cassette) transporters, which facilitate the transport of natural metabolites and xenobiotics—such as antifungal compounds—through ATP hydrolysis, playing a key role in antifungal resistance [55]. Moreover, *E. weberi* and *U. virens* demonstrated a marked reduction in cytochrome P450 enzymes. Conversely, *S. chartarum* and *S. elegans* demonstrate considerable expansion in genes linked to the MFS_1 domain, but both *S. elegans* and *P. chlamydosporia* indicate an enrichment of genes pertaining to cytochrome P450. The expanded gene sets in these species are also enriched in Pfam domains such as HET, adh short, and Ank, which are associated with functions including heterokaryon incompatibility–mediated cell death [56], short-chain dehydrogenase/reductase–mediated primary metabolism [57], and protein–protein interaction scaffolding in signaling or developmental pathways [58].

### 3.4. GPCRs in Biosynthetic Gene Clusters

Furthermore, given that Pth11-like GPCRs, which detect cellulose and plant cell wall components, trigger responses promoting fungal infection [24,59], we investigated their association with key proteins involved in secondary metabolism across multiple species. In a total of 212 species, 327 Pth11-like GPCRs are positioned within biosynthetic gene clusters (BGCs) that participate in secondary metabolism, comprise 4.2% of all Pth11-like GPCRs. These GPCRs are located most frequently in close proximity to polyketide synthase (PKS) and nonribosomal peptide synthetase-like (NRPS-like) genes (Supplementary File S1 Table S4), which implies that they may play a role in the regulation of secondary metabolite production. In addition to Pth11-like GPCRs, Class 12 GPCRs include 41 receptors (4.1% of all Class 12 sequences) located within BGCs, most commonly positioned near PKS genes. Other GPCR classes contribute fewer receptors to BGCs, and seven classes (Classes 1, 4, 7, 10, 11, 16, and 17) have no GPCRs located within BGCs.

### 3.5. Novel GPCRs Predicted Using Machine Learning

**Figure 4:**
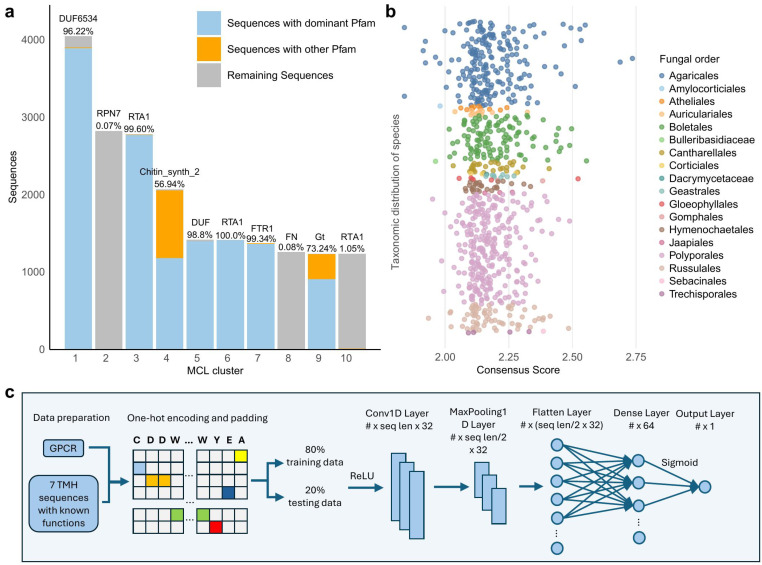
Identification of novel GPCRs. (**a**) Top ten largest MCL clusters for sequences with seven TMHs, excluding the 28,294 sequences comprising newly identified GPCRs and those with seven TMHs but invalid topologies. The blue portion of each bar represents the number of sequences with the dominant Pfam domain in that cluster, the orange portion represents sequences with other Pfam domains, and the gray portion represents the remaining sequences. The dominant Pfam domain and its percentage are displayed on top of each bar. FN in cluster 8 refers to Flavi_NS4A and Gt in cluster 9 refers to Glyco_trans_2_3. (**b**) Consensus score distribution (sum of self-built CNN, ProteInfer, and DeepFRI models) across 222 species in 18 fungal orders. (**c**) Architecture of the self-built 1D-CNN model. Newly identified 12,587 GPCR sequences with GPCR-related Pfam domains are used as the positive dataset, while 18,221 sequences with seven TMHs in MCL clusters dominated by Pfam domains unrelated to GPCR functions are used as the negative dataset. Sequences are transformed into one-hot encoding and padded. 80% of sequences are used as training data, and 20% as testing data. The sequences are input into a convolutional 1D layer with 32 filters, a kernel size of 3, and ReLU activation. This is followed by a max-pooling layer with a pool size of 2. The flattened output is passed through a fully connected layer with 64 neurons (ReLU activation), and finally, a sigmoid-activated output layer is used for binary classification.

After excluding sequences previously identified as putative GPCRs by HMM-based searches, 141,666 protein sequences, containing seven TMHs, remain unidentified as GPCRs among 1357 fungal species. MCL clustering of these sequences resulted in 1969 clusters, with an average size of 71.5 sequences, and the biggest cluster of 4046 sequences. Pfam domain identification was conducted on the ten largest clusters to elucidate the functional roles of the sequences. The analysis indicated that the majority of sequences within specific clusters have identical Pfam domains, whilst other clusters predominantly lacked identifiable Pfam domains, indicating proteins of unknown functions (Figure 4a). Given that 96.22% of the sequences within the largest cluster contained the DUF6534 domain, we suggest that these sequences might exhibit GPCR functionality, potentially representing a new class of GPCRs.

To test this hypothesis, machine learning algorithms ProteInfer and DeepFRI were employed to determine whether the 3893 sequences containing the DUF6534 domain in the largest MCL group are potential novel GPCRs. ProteInfer predicted 1793 sequences with GPCR activity with a confidence score exceeding 0.8 and within them, 1637 sequences were predicted to belong to the GPCR_A Pfam clan, which includes numerous members of the rhodopsin superfamily. Whereas DeepFRI predicted 2204 sequences to have GPCR activity and only 25 of them with a confidence score above 0.5 (considered high confidence), some sequences with DeepFRI scores <0.2 were correct predictions [60]. The substantial divergence between the outcomes of ProteInfer and DeepFRI indicates a need for a novel classifier to assess the GPCR activity of proteins containing the DUF6534 domain.

Consequently, we designed a one-dimensional convolutional neural network (CNN) model designed to detect putative new GPCRs among proteins anticipated to possess 7–9 TMHs (Figure 4c). We evaluated 8 or 9 TMHs in addition to 7 TMH proteins because Class 11 GPCRs have 9 TMHs [23], while also considering possible discrepancies in the TMHMM prediction. The model was trained on 12,587 sequences from 14 GPCR classes, as well as a negative dataset of 18,221 sequences that featured 7 TMHs but were linked to non-GPCR functions (see Methods for details). An F1 value of 0.97 was obtained during the model validation process, indicating exceptional performance in the classification of GPCRs and non-GPCRs. Utilizing this model and filtering out sequences with inappropriate topologies, we predicted 69.9% of sequences within the largest clusters as GPCRs. Subsequently, we integrated confidence scores from ProteInfer (scores > 0.8), DeepFRI (scores > 0.2), and the CNN model (scores >0.9) to derive a consensus prediction for GPCR activity. As a result, consensus GPCR prediction was obtained for 767 sequences from 222 fungal species, all in the Agaricomycotina subphylum in Basidiomycota (Figure 4b). These species included *Dioszegia hungarica* from Tremellomycetes and *Calocera cornea* from Dacrymycetes and the rest from Agaricomycetes. The orders Polyporales, Agaricales, and Boletales contain the most sequences. Notably, Agaricales has the highest number of sequences with scores above 2.50. Since most consensus scores range between 2 and 2.50, establishing a threshold for accurate prediction of a novel GPCR is challenging. Therefore, AlphaFold Multimer [35] was used to evaluate the interaction potential between the predicted GPCRs and the G-alpha proteins to in silico validate their function.

### 3.6. Predicted Interactions Between Potential Novel GPCRs and G-Alpha Proteins

**Figure 5:**
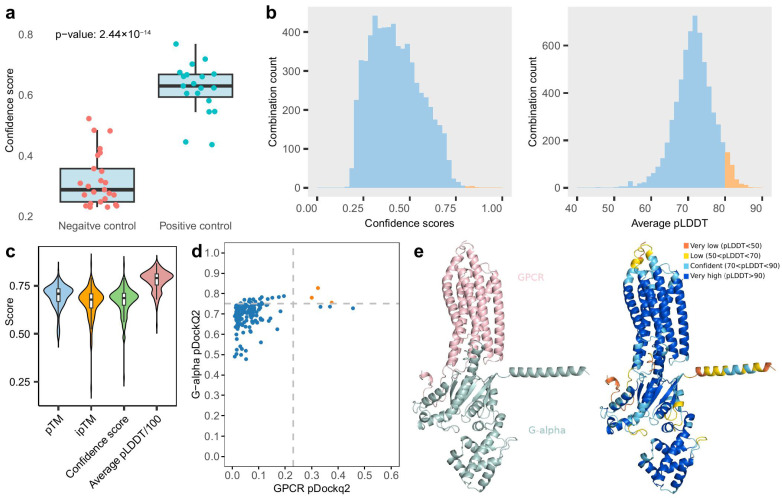
Evaluation of GPCR–G-alpha interactions with AlphaFold Multimer. (**a**) Comparison of AlphaFold Multimer confidence scores between interacting and non-interacting protein pairs [35]. For each combination, five AlphaFold Multimer models were generated and the highest scores are presented. The median, 25th, and 75th percentiles are shown. (**b**) Distribution of confidence scores (calculated as 0.8 × iPTM + 0.2 × PTM) and the average pLDDT score across all GPCR and G-alpha combinations. Combinations with a score higher than 75 are highlighted in orange. Similarly, combinations with an average pLDDT score above 80 are considered high-confidence and are also highlighted in orange. (**c**) Evaluation metrics for the top pair from 155 species, based on four AlphaFold Multimer results. (**d**) pDockQ2 scores for both GPCR and G-alpha are shown. Interactions with a minimum pDockQ2 score greater than 0.23 are considered high-confidence. (**e**) The structure of the GPCR and G-alpha interaction with the highest confidence, as predicted in *Galerina marginata*. The corresponding pLDDT scores of the structure are also displayed.

After identifying the most promising novel GPCRs across 222 species, the G-alpha proteins were quantified for each species, showing considerable variation in their quantities. However, there is no clear relationship between the quantity of G-alpha proteins and the total number of GPCRs (Pearson correlation = −0.256). To identify interactions between novel GPCRs and G-alpha proteins using AlphaFold Multimer [35], we conducted preliminary tests with known functional pairs and mismatched counterparts. There are two well-characterized GPCR–G-alpha protein pairs involved in distinct pathways controlling mating in *S. cerevisiae*. The first pair, related to nutrient availability, involves Gpr1 and Gpa2 [11]. Gpr1 functions as a carbon receptor sensing glucose, causing a conformational change that activates the G-alpha protein Gpa2. The second pair involves Ste2 interacting with Gpa1 in the pheromone sensing pathway [61]. Leveraging published research, we note that Gpa1 and Gpa2 are the only two Gα subunit genes in the *S. cerevisiae* genome responsible for distinct pathways [62]. Additionally, 17 well-characterized GPCR–G-alpha protein pairs in two other fungal species (*C. albicans*, and *Cryptococcus neoformans*), while one plant species (*Arabidopsis thaliana*) and human were included as positive control (Supplementary File S1 Table S5). Since Gpa1 can productively couple with the α-factor receptor Ste2, whereas Gpa2 cannot [63], we exchanged the G-alpha proteins between the GPCRs to create two pairs as negative controls, along with 23 extra pairs composed of non-GPCR proteins (with unrelated Pfam domains) from *S. cerevisiae* paired with Gpa1 or Gpa2 as negative controls. AlphaFold Multimer was conducted for these 44 protein pairs. The ACM2-GNAI2 pair in humans achieved the highest confidence score of 0.77 (0.8 ipTM + 0.2 pTM) [35]. In fungi, the highest score was 0.70 for the Ste2-Cag1 pair from *Candida albicans*. In contrast, the best score among the non-interacting pairs was 0.52. On average, interacting pairs had a mean confidence score of 0.62, significantly higher than the mean score of 0.32 for non-interacting pairs (Figure 5a). A two-sided *t*-test revealed that AlphaFold Multimer scores are significantly different between interacting and non-interacting complexes (*p* = 2.44 × 10^−14^). This demonstrates AlphaFold Multimer’s strong performance in distinguishing between interacting and non-interacting complexes.

However, the scores for some interacting complexes are moderate for known interactions but lack detailed information, as they do not include per-residue local confidence (pLDDT) [52] provided by AlphaFold 2 or the global confidence measure provided by the predicted aligned error (PAE) in AlphaFold Multimer. Therefore, we implemented the pDockQ2 score [51], which is capable of assessing AlphaFold Multimer results that incorporate per-residue and PAE information. pDockQ2 scores of 0.262 and 0.394, respectively, were obtained for Gpr1 and Gpa2, with scores exceeding 0.23 suggesting high-confidence interactions. The scores of Ste2 and Gpa1 in the second pair were 0.307 and 0.409, respectively. The effectiveness of the pDockQ2 score in identifying protein interactions was underscored by the unambiguous distinction of the pDockQ2 scores for the non-interacting pairs, with 0.022 for Gpr1-Gpa1 and 0.021 for Ste2-Gpa2.

Then, we employed AlphaFold Multimer to assess all 6376 possible pairings of new GPCRs and G-alpha proteins across 155 fungal species. By choosing the maximum confidence level for each combination, we determined that the overall average confidence score is 0.425. We identified 1872 combinations with confidence scores greater than 0.5 across 150 of the 155 species (Supplementary File S1 Table S6), indicating good overall model quality [64]. This corresponds to an average of 12.48 combinations per species. Among all species, *Galerina marginata*, a white-rot fungus in the Cortinariaceae family, shows the highest number of high-confidence combinations (51, 2.73% of all combinations). In addition, six other species, including *C. anzutake*, *Armillaria gallica*, *Sphaerobolus stellatus*, *Ceriporiopsis (Gelatoporia) subvermispora*, *Fibulorhizoctonia psychrophila*, and *Antrodia serialis*, each also exhibit more than 2% of the total combinations, all belonging to the class Agaricomycetes. Significantly, merely nine combinations possess confidence levels above 0.8, so categorizing them as very high-confidence predictions. All nine come from *G. marginata*. The mean pLDDT for all combinations is 71.5, with 312 combinations exhibiting pLDDT values over 80 (Figure 5b), suggesting possible interactions between the new GPCRs and G-alpha proteins.

There was considerable variation in the highest confidence scores for different combinations within a single species (Supplementary File S1 Table S6). We selected the combination with the highest confidence score for each species and summarized their evaluation metrics. The pTM and ipTM have similar average scores with ipTM having larger variance (Figure 5c). Majority of the average pLDDT concentrated at 80, which can be considered as high confidence. Then we calculated the pDockQ2 score for the species. Six species exhibited a minimum pDockQ2 > 0.23, and three of these had a pTM > 0.75 (Figure 5d), indicating very high-confidence combinations. The three species identified in this study are *G. marginata* (Figure 5e), *Bjerkandera adusta*, a known plant pathogen that causes white rot in both live trees and dead wood, and *Coprinopsis* sp. MPI-PUGE-AT-0042, which colonizes the roots of healthy *A. thaliana* plants grown in natural soil after surface sterilization. All three species belong to the Agaricomycetes class.

We further applied AlphaFold Multimer to GPCR–G protein heterotrimer combinations to examine whether including G-beta and G-gamma subunits could improve interaction identification. We ran 411 possible pairings of GPCR and G protein heterotrimer across 10 species (Supplementary File S1 Table S7), selecting those with the highest confidence scores from the GPCR–G-alpha protein combinations. Among these, 246 combinations showed confidence scores greater than 0.5 (Supplementary File S1 Table S7). Within this set, 57 combinations had very high confidence scores (>0.75) across 9 of the 10 species, with the exception of *Gloeophyllum trabeum*, with 31.6% of combinations from *C. anzutake* and 21% from *G. marginata*. The 3D structure of the GPCR–G protein heterotrimer with the highest confidence score in each of the nine species is shown in Supplementary Figure S1. The average pLDDT was 76.9, with 91 combinations exceeding 80. These performance metrics and high-confidence combinations are similar to those observed in previous GPCR–G-alpha protein interaction predictions, providing more robust support for their use in future wet-lab validation. Furthermore, we calculated the pDockQ2 score for all combinations. Only one combination from *C. anzutake* exhibited a minimum pDockQ2 > 0.23 (Supplementary File S1 Table S8), highlighting the strictness of this metric in retaining only very high-confidence interactions. This indicates that pDockQ2 imposes an even more stringent filter than used for GPCR–G-alpha protein predictions, as all four sequences (GPCR plus the three G protein subunits) must surpass the threshold of 0.23.

## 4. Discussion

This study provides a comprehensive analysis of GPCR diversity and distribution across more than 1300 fungal species, integrating the 14 established fungal GPCR classes with three additional mammalian-like classes not previously incorporated into the fungal GPCR classification. We observed substantial variation in GPCR abundance and distribution across Ascomycota, Basidiomycota, and EDF. Several EDF groups, such as Olpidiomycotina, Apansporoblastina, and Pansporoblastina which encompass parasitic fungi, lacked any representatives of 17 recognized GPCR classes. This suggests that some EDF lineages may have bypassed the evolutionary development of GPCR-mediated signaling pathways, instead possibly relying on alternative membrane proteins or signaling mechanisms. Such lineage-specific losses likely reflect a diminished dependence on GPCRs as a result of ecological specialization or variations in membrane structure, emphasizing the divergent evolutionary pressures on GPCR evolution.

Among the major fungal groups, Ascomycota exhibited the highest overall GPCR abundance and diversity, particularly with the dominance of Pth11-like GPCRs (Class 14). Class 1 GPCRs were exclusive to Ascomycota, suggesting that this class evolved after the divergence of Ascomycota from EDF. Ascomycota either developed these receptors independently or expanded an ancestral signaling pathway into the Class 1 GPCRs. Their absence in Basidiomycota implies replacement by alternative pheromone signaling mechanisms. In support of this, Basidiomycota, particularly Agaricomycota, showed a predominance of Class 2 GPCRs. Additionally, the absence of GPCR classes 5, 6, and 7 in Basidiomycota, despite their presence in Ascomycota and EDF, potentially reflects redundancy or shifts in environmental adaptation.

The functional relevance of the 14 classes of identified GPCRs is confirmed by presence of GPCR-related Pfam domains in over 98.6% of sequences with Pfam domains. Interestingly, the CFEM domain, commonly associated with pathogenicity, was found in a small subset of class 14 GPCRs, implying that it is not required for Pth11-like GPCR function. Notably, Pth11-like GPCRs make for almost one-third of all discovered GPCRs, and they are all found exclusively in Pezizomycotina, consistent with prior studies [29] but confirmed here for a much larger number of genomes.

Furthermore, we identified 2089 mammalian GPCR homologs across 594 fungal species, thereby expanding the distribution of three mammalian-like classes (Rhodopsin, Glutamate, and Frizzled). Although reported previously, these classes are not commonly included in fungal GPCR classification system. We demonstrate their wider presence in fungi, and expand GPCR classification to now include 17 classes. Unlike the 14 classes enriched in Ascomycota, mammalian GPCR homologs are predominantly concentrated in EDF lineages, with Rhodopsin (newly defined as Class 15) concentrated in Entomophthoromycotina, while Glutamate (Class 16) prominently abundant in both Blastocladiomycota and Chytridiomycota. This pattern suggests a shared ancestral GPCR repertoire between EDF species and mammals. Meanwhile, fungi have evolved numerous lineage-specific GPCRs, likely due to adaptations to a variety of environmental conditions, leading to significant reductions in the previously recognized 14 GPCR classes in EDF species. These discoveries underscore the evolutionary plasticity of the GPCRs within fungal lineages and emphasize the importance of ecological and evolutionary forces in diversifying signaling systems in fungi.

Our analysis further indicates considerable diversity in the prevalence of Pth11-like GPCRs among over 600 species in Pezizomycotina. We found that variations in the abundance of these receptors were closely associated with gene duplication and loss events, along with broader genomic dynamics, including the expansion and contraction of gene families. Species with abundant Pth11-like GPCRs, such as *P. chlamydosporia* and *S. elegans*, demonstrated an increased number of gene family expansions, implying a possible correlation between receptor prevalence and genomic dynamics. Conversely, species with a few Pth11-type GPCRs, such as *E. weberi*, exhibited a greater degree of gene family contraction.

Functional annotations using Gene Ontology (GO) and Pfam domain enrichment analyses suggest a link between Pth11-like GPCRs, secondary metabolism, and host–pathogen interactions. Species with elevated counts of Pth11-like GPCRs exhibit an expansion of Pfam domains such as MFS_1 and cytochrome P450, suggesting enhanced capabilities for environmental adaptation, nutrient acquisition, and stress response. For example, *P. chlamydosporia* demonstrates a multitrophic lifestyle, encompassing parasitism on nematode eggs, soil saprophytism, and endophytic colonization of plant roots [65], reflecting its ability to adapt to diverse environments. In addition, *S. elegans* evolved an expanded repertoire of signaling-related genes, exhibits narrow host specificity targeting *Rhizoctonia solani* AG-3, suggesting that the expansion of Pth11-like GPCRs may reflect regulatory complexity rather than an expanded host range. This observation contradicts previous studies which associated higher quantities of Pth11-like GPCRs with broader host specificity [66]. Conversely, species with fewer Pth11-like GPCRs show a significant reduction in MFS_1 and cytochrome P450 domains, indicating notable differences in metabolic capacities and ecological strategies. *E. weberi*, a specialized parasite, has undergone a reduction in its GPCR repertoire within a streamlined genome, relying on its host for metabolic requirements. This reliance likely drove its genome reduction [53] and simplified host interaction strategies. Furthermore, in 212 species, 4.2% of Pth11-like GPCRs were found adjacent to biosynthetic gene clusters, particularly those encoding polyketide synthases (PKS) and nonribosomal peptide synthetases (NRPS). This spatial association implies a regulatory role for Pth11-like GPCRs in secondary metabolism. Besides, Class 12 GPCRs exhibit a comparable proportion of sequences located within BGCs, and several additional GPCR classes show this pattern as well, although with fewer representatives. Their proximity to biosynthetic genes suggests that the associated metabolites may serve as candidate ligands for these GPCRs during experimental characterization of these receptors’ signaling functions.

Additionally, we identified 767 novel GPCRs across 222 fungal species, with consensus predictions of GPCR activity facilitated by integrating multiple machine learning methods: ProteInfer, DeepFRI, and a custom CNN model. Many of these sequences belong to an uncharacterized GPCR family featuring the DUF6534 domain. These novel GPCRs were especially enriched in the orders Polyporales, Agaricales, and Boletales (Agaricomycotina, Basidiomycota). Despite the discrepancies between ProteInfer and DeepFRI, these novel GPCR candidates may represent a previously unrecognized class of signaling proteins.

To explore their functional potential, we used AlphaFold Multimer to predict interactions between novel GPCRs and G-alpha proteins across 155 fungal species. In our analysis of 19 well-characterized GPCR–G-alpha protein pairs across species, we found that combining AlphaFold Multimer and pDockQ2 scores reliably distinguishes true interactions. Out of the 6376 combinations that were assessed, nine high-confidence GPCR–G-alpha protein interactions were identified, all from *G. marginata*. Additional strong predictions were obtained for *B. adusta* and *Coprinopsis* sp. This provides certain validation that these novel GPCRs may have a potential function in fungal signaling pathways to be followed up with experimental validation. It is important to note that the high-confidence interactions predicted in *G. marginata*, *B. adusta*, and *Coprinopsis* sp. indicate that these GPCRs may be essential for plant–fungal interactions, particularly in the processes of wood decomposition and root colonization. Structural metrics of AlphaFold Multimer (pTM/ipTM, pLDDT, and pDockQ2) support the candidate nature of the predicted GPCR–G-alpha pairs and suggest directions for further experimental investigation. In addition, examining GPCRs located near BGCs may help identify potential ligands for experimental validation of their function.

Our analysis of more than 1300 fungal genomes highlights the extraordinary diversity of GPCRs across fungal tree of life. We have expanded the fungal GPCR classification to 17 classes by adding three classes from mammalian classification and predicting novel GPCR-related domains. In addition, we report that Pth11-like GPCR abundance varies significantly among species in Pezizomycotina, primarily due to both genomics dynamics and ecological specialization. Our work sets a stage for future experimental validation and functional characterization of GPCRs in fungal signaling and pathogenesis.

## Figures and Tables

**Table 1:** List of all fungal GPCR classes, related Pfam domains, and their functions. The four mammalian GPCR classes detected in fungi are highlighted in bold.

GPCR Class	Pfam Domain	Domain Function in Pfam	Pfam ID
1	STE2	Fungal pheromone mating factor STE2 GPCR	PF02116
2	STE3	Pheromone A receptor	PF02076
3	Git3	G protein-coupled glucose receptor regulating Gpa2	PF11710
4	PQ-loop	PQ-loop repeat	PF04193
5	Dicty_CAR	Slime mold cyclic AMP receptor	PF05462
6	RGS	Regulator of G protein signaling domain	PF00615
**7**	**7tm_2**	**7 TM receptor (Secretin-like family)**	**PF00002**
8	HlyIII	Haemolysin-III related	PF03006
9	Bac_rhodopsin	Bacteriorhodopsin-like protein	PF01036
10	Lung_7-TM_R	Lung seven transmembrane receptor	PF06814
11	ABA_GPCR	Abscisic acid G-protein coupled receptor	PF12430
12	DUF3112	Protein of unknown function (DUF3112)	PF11309
13	Solute_trans_a	Organic solute transporter Ostalpha	PF03619
14	CFEM	CFEM domain	PF05730
**15**	**7tm_1**	**7 TM receptor (Rhodopsin family)**	**PF00001**
**16**	**7tm_3**	**7 TM sweet-taste receptor of 3 GCPR (Glutamate family)**	**PF00003**
**17**	**Frizzled/Fz**	**Frizzled family membrane region (Frizzled family)**	**PF01534/PF01392**

**Table 2:** Pth11-like GPCR characteristics in twelve selected species in Pezizomycotina. Twelve selected species are arranged in ascending order of Pth11-like GPCR abundance. The number of Pth11-like GPCR duplication and loss events, identified using Notung [44], along with the number of expanded and contracted gene families in their genomes, as determined by CAFE [45], are displayed.

Species	MycoCosm Portal ID	Pth11-like GPCR Abundance	Gene Duplications	Gene Losses	Expanded Gene Families	Contracted Gene Families
*Escovopsis weberi*	Escweb1	1	0	14	115	2816
*Ophiocordyceps camponoti-rufipedis*	Ophca1	2	0	1	113	617
*Ophiocordyceps kimflemingae*	Ophun1	2	0	1	162	175
*Ustilaginoidea virens*	Ustvir1	2	0	11	99	3361
*Tolypocladium capitatum*	Tolca1	3	0	2	222	2400
*Metarhizium brunneum*	Metbr1	6	0	4	89	451
*Metarhizium robertsii*	Metro1	6	0	4	179	138
*Metarhizium guizhouense*	Metgui1	8	0	4	265	433
*Stachybotrys chartarum*	Stach1	12	0	3	343	484
*Stachybotrys chlorohalonata*	Stachl1	12	0	3	136	560
*Pochonia chlamydosporia*	Pocchl1	13	1	2	869	742
*Stachybotrys elegans*	Stael1	24	4	7	1074	539

## Data Availability

The query sequences of all 14 classes of GPCRs were sourced from previous literature [67]. The genomes of 1357 fungi species are publicly available in MycoCosm [36]. Supplementary File S2 contains all 28,294 GPCRs classified into 17 classes. Phylogenetic tree of the Pezizomycota was obtained from the MycoCosm portal [36].

## Supplementary Materials

The following supporting information can be downloaded at: https://www.mdpi.com/article/10.3390/jof12010030/s1.
