# Supplementary material for: Identification and Classification of Fungal GPCR Gene Families
Source: J Fungi (Basel). 2025 Dec 30;12(1):30. doi: 10.3390/jof12010030 (PMC12842992; doi:10.3390/jof12010030)
Supplement: Supplementary file 1 [file jof-12-00030-s001.zip › Supplementary Figure S1.pdf]

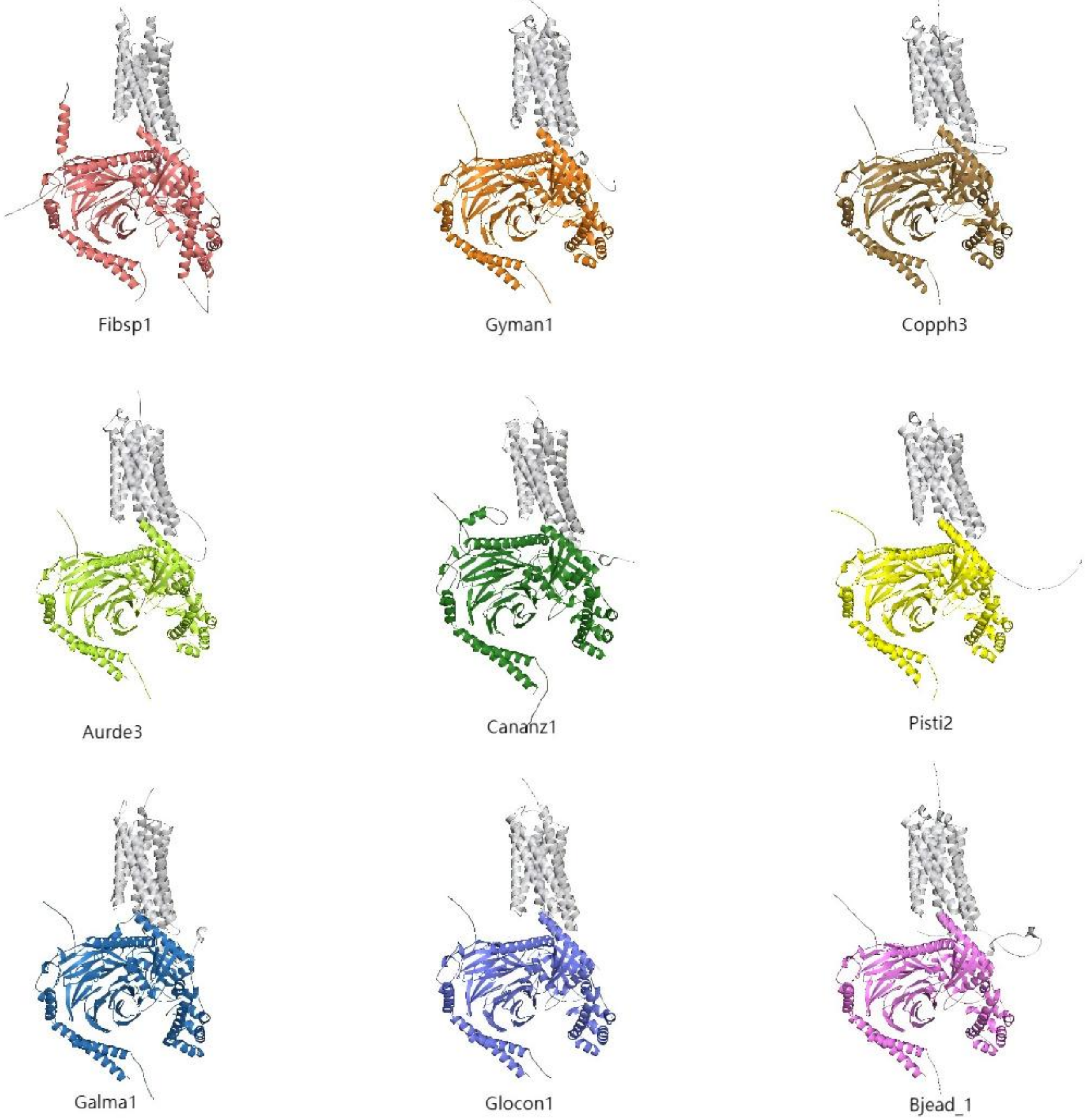

Supplementary Figure S1. The 3D structures of the GPCR-G protein heterotrimer with the highest confidence score predicted by AlphaFold-Multimer in each of the nine species. Fibsp1, Gyman1, Copph3, Aurde3_1, Cananz1, Pisti2, Galma1, Glocon1, and Bjead1_1 represent *Fibulorhizoctonia psychrophila CBS 109695; Gymnopus androsaceus JB14; Coprinopsis sp. MPI-PUGE-AT-0042; Auricularia subglabra; Cantharellus anzutake C23; Pisolithus tinctorius Marx 270; Galerina marginata; Gloeopeniophorella convolvens OM19405; and Bjerkandera adusta,* respectively.
